# Global surface features contribute to human haptic roughness estimations

**DOI:** 10.1007/s00221-021-06289-0

**Published:** 2022-01-16

**Authors:** Huazhi Li, Jiajia Yang, Yinghua Yu, Wu Wang, Yulong Liu, Mengni Zhou, Qingqing Li, Jingjing Yang, Shiping Shao, Satoshi Takahashi, Yoshimichi Ejima, Jinglong Wu

**Affiliations:** 1 Graduate School of Interdisciplinary Science and Engineering in Health Systems, Okayama University, 3-1-1 Tsushima-Naka, Kita-ku, Okayama, 700-8530 Japan; 2 Section On Functional Imaging Methods, National Institute of Mental Health, Bethesda, MD USA; 3 School of Mechatronical Engineering, Beijing Institute of Technology, Beijing, China; 4 School of Psychological and Cognitive Sciences, Peking University, Beijing, China; 5 Department of Teacher Education, Wenzhou University, Wenzhou, China; 6 School of Computer Science and Technology, Changchun University of Science and Technology, Changchun, China; 7 School of Social Welfare, Yonsei University, Seoul, Korea

**Keywords:** Haptic roughness perception, Raised-dot surface, Local feature, Global feature

## Abstract

Previous studies have paid special attention to the relationship between local features (e.g., raised dots) and human roughness perception. However, the relationship between global features (e.g., curved surface) and haptic roughness perception is still unclear. In the present study, a series of roughness estimation experiments was performed to investigate how global features affect human roughness perception. In each experiment, participants were asked to estimate the roughness of a series of haptic stimuli that combined local features (raised dots) and global features (sinusoidal-like curves). Experiments were designed to reveal whether global features changed their haptic roughness estimation. Furthermore, the present study tested whether the exploration method (direct, indirect, and static) changed haptic roughness estimations and examined the contribution of global features to roughness estimations. The results showed that sinusoidal-like curved surfaces with small periods were perceived to be rougher than those with large periods, while the direction of finger movement and indirect exploration did not change this phenomenon. Furthermore, the influence of global features on roughness was modulated by local features, regardless of whether raised-dot surfaces or smooth surfaces were used. Taken together, these findings suggested that an object’s global features contribute to haptic roughness perceptions, while local features change the weight of the contribution that global features make to haptic roughness perceptions.

## Introduction

People can extract both geometric (e.g., curvature) and material properties (e.g., roughness and compliance) (Whitaker et al. 2008) to form a representation of objects by touch. As one of the most salient perceptions of surface material properties, haptic roughness perception has attracted the attention of many researchers (see Tiest 2010 for review). For example, many researchers have studied roughness perception on coarse surfaces by modulating local physical features, such as the groove width of the grating surface and dot spacing of raised-dot surfaces (Dépeault et al. 2009; Drewing 2016; Lawrence et al. 2007; Sutu et al.^2013^). These studies indicated that the spatial period of the surface plays a crucial role in roughness perception. However, the psychophysical function between the spatial period and perceived roughness is also modulated by other properties, such as the shape and height of elements (Drewing 2016; Goodman and Bensmaia 2017; Sutu et al. 2013). Thus, roughness perception is a complex, multidimensional sensation that is dependent on the combination of different spatial and temporal factors of a surface (Yang et al. 2017). Moreover, both spatial and temporal properties depend not only on local features on the surface (e.g., dot spacing) but also on its surface global features (e.g., curved surface). To date, however, the way in which global features affect haptic roughness perception remains unclear.

Haptic roughness perception is highly dependent on hand exploration (Hollins and Risner 2000). People can adjust their hand movements during object exploration to achieve the best perception based on the features of the stimulus, such as lateral motion for roughness and contour-following for shape (Lederman and Klatzky 1987). While this occurrence is almost the case in daily life, people can extract information about different dimensions simultaneously (Metzger et al. 2019; Mueller et al. 2019) and integrate them into one representation (Lacey et al. 2010; Lederman et al. 1993). For example, individuals perceived a smooth cube as being significantly larger than a rough cube of the same physical volume (Tiest et al. 2012). Thus, different physical properties interact with each other to form object representations in the brain (Tiest 2010). Moreover, touch is a composite perception that relies on the responses of large numbers of different receptors (Saal and Bensmaia 2014). Pertinently, information on local features depends on cutaneous input, while proprioceptive input provides information on global features; people obtain constant perception by integrating both types of input (Yoshioka et al. 2011). Therefore, it is reasonable to assume that haptic roughness perception is affected by integrating local and global features in an appropriate exploratory procedure.

Spatial and temporal information, which is important for forming roughness perception, is encoded by different afferents. Slowly adapting cutaneous afferents (SAs) dominate the coding for spatial information, which includes not only the spatial map of an object, such as the dot distance of a raised-dot surface (Hollins and Bensmaïa 2007; Weber et al. 2013) but also the curvature on the order of the size of a fingertip (Birznieks et al. 2001; Goodwin et al. 1995, 1997; Goodwin and Wheat 2004; Pruszynski and Johansson 2014). Furthermore, the temporal information (texture-specific vibration) coded by rapidly adapting (RA) receptors plays an important role in roughness coding (Fagiani and Barbieri 2016; Weber et al. 2013), and direct skin contact is not necessary for the coding of temporal information (Johnson et al. 2002; Yoshioka et al. 2011). In most cases, SAs and RA receptors simultaneously contribute to the coding of roughness. Although we cannot strictly distinguish the contributions of different nerves in a behavioral experiment, it is possible to reduce the contribution of SA receptors using indirect touch during roughness perception. This approach allows us to explore how the peripheral nervous system contributes to the interaction of global and local features in roughness perception.

In the present study, we used the same series of haptic stimuli that combines local features (raised-dot spacing) and global features (cycle number of the curved surface) that was used in our previous study (Yang et al. 2021a, b) to investigate haptic roughness perception. A total of 25 three-dimensional (3D) printed stimuli (five kinds of curves × five varieties of dot spacing) were made by modulating these two features. Participants were asked to explore the surface of each stimulus and then estimate the roughness of the surface. Exploration was performed in a different fashion in a series of experiments. This paradigm design allowed us (1) to investigate how the roughness estimation correlated with the local and global changes, (2) to test the interaction between local and global features on roughness estimation and to evaluate whether the effect of global features was stable after changing hand motions, (3) to elucidate the underlying mechanism of the interaction between local and global features on roughness estimation by indirectly using surface exploration with a rigid probe, (4) to investigate the effect of the curvature of a single curve on roughness estimation during static touch, and (5) to eliminate the possibility that variation of dot spacing in different parts of the curved surface would influence roughness estimation.

## Method

### Participants

Twenty-three right-handed participants aged between 22 and 35 years (mean ± SD = 26.5 ± 3.7, sex, 20 males and three females) volunteered for the present experiment. Six individuals participated in all experiments in the present study (Experiments 1–5). Nine individuals participated in 4 experiments (six for Experiments 2–5 and three for Experiments 1, 3, and 5). Two individuals participated in 3 experiments (one for Experiments 2, 3, and 4 and one for Experiments 1, 2, and 5). Two individuals participated in 2 experiments (Experiments 1 and 2). Four individuals only took part in Experiment 1. Each experiment included sixteen participants, and the order effect across experiments was counterbalanced by the Latin square method. All were naive to the purpose of these experiments that were being conducted. All participants were healthy and reported no history of neurological or psychiatric disorders. All participants provided written informed consent in compliance with the policies of the local Medical Ethics Committee of Okayama University. The testing procedures were reviewed and approved by the local Medical Ethics Committee of Okayama University.

### Stimuli

#### Coarse surface

**Fig. 1:**
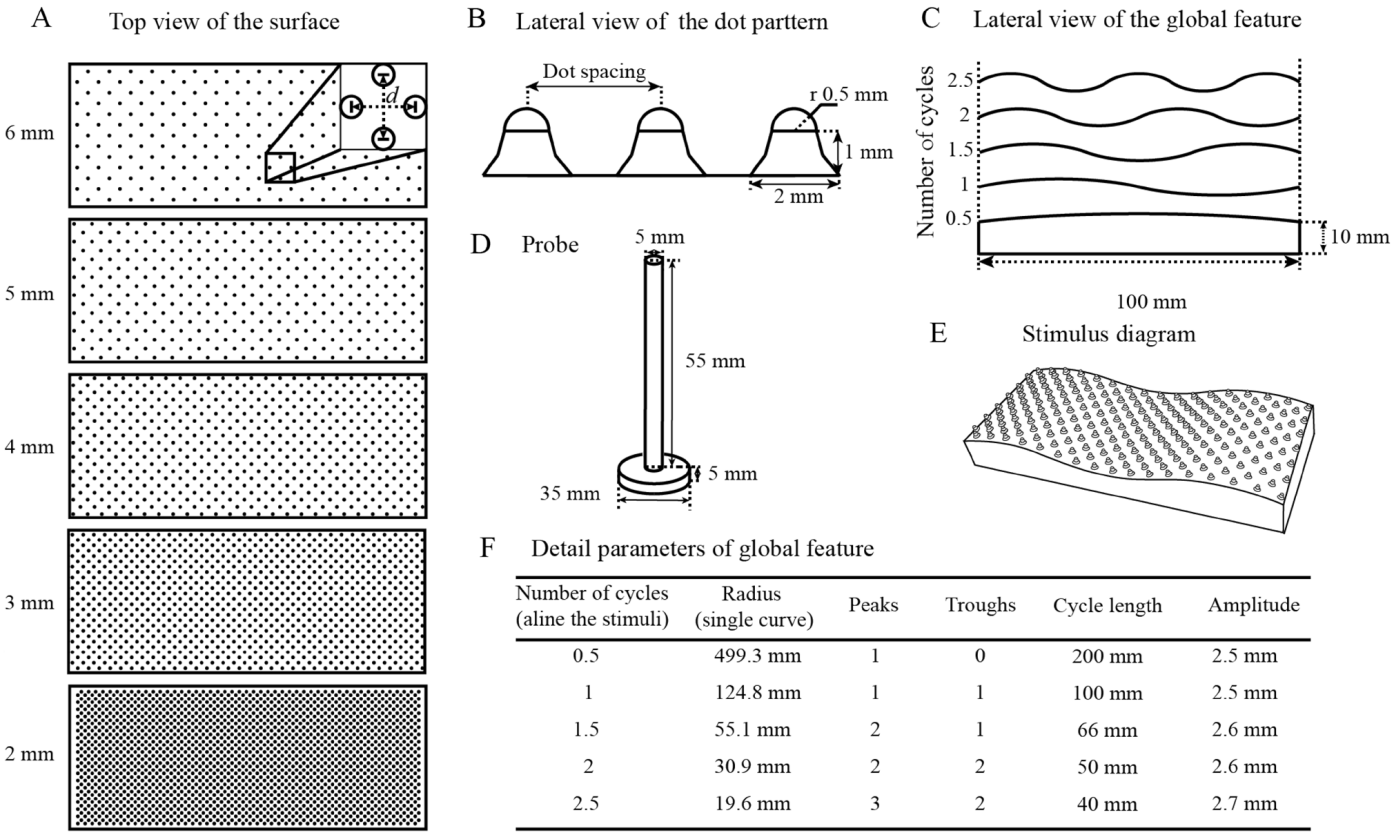
Stimuli configuration. **A** Schematic representation of the raised-dot surfaces. The length of the dotted line (*d*) of the enlarged image represents the parameter of dot spacing. **B** The parameters of a single dot and dot spacing were measured between the centers of adjacent dots. **C** Schematic representation of the 3D contour of the surface, which consisted of 0.5–2.5 cycles. **D** Schematic representation of the probe used in Experiment 3. **E** One example stimulus (reference in Experiment 1) with 1.5 cycles and 4 mm dot spacing. **F** Parameters of the global feature

A series of coarse stimulus sets combining local features (i.e., raised-dot texture) and global features (i.e., curved surface), which were changed in a parametric manner, were used in Experiments 1–4 (Yang et al. 2021a, b). The texture of stimuli that changed on the order of millimeters was defined as a local feature, while the global feature of the surfaces changed on the order of centimeters. All stimuli (length: 100 mm, width: 40 mm) were printed with acrylonitrile butadiene styrene (ABS) by a 3D printer. Figure 1 shows the detailed parameters of the coarse stimuli and the probe used in Experiment 3 (Fig. 1D). The stimuli were cuboids with a curved top-side surface on which raised dots were arranged in a square pattern (Fig. 1A). Specifically, Fig. 1a shows five kinds of textured surfaces. The raised dots had a height of 1.5 mm and were formed by a hemisphere (radius 0.5 mm) superimposed on the top of the cylinders (Fig. 1B). Raised dots were arranged diagonally on the curved surface, and each square had identical dot spacing (distances between the centers of adjacent the dots) in the longitudinal direction. Dot spacing ranged from 2 to 6 mm (increments of 1 mm). Then, five kinds of raised dots were printed on the tops of five kinds of curved surfaces (Fig. 1C), in which the cycle length changed from 200 mm (0.5 cycles running along the stimulus) to 40 mm (2.5 cycles running along with the stimulus) with the radius of a single curve ranging from 499.3 mm (0.5 cycles) to 19.6 mm (2.5 cycles). Figure 1E presents one example stimulus (dot spacing: 4 mm; number of cycles: 1.5) that was used as a reference in Experiment 1. The detailed parameters of the global feature are presented in Fig. 1F. Combined with each level of both features, 25 stimuli were obtained in total. These stimuli ensured that participants could perform a haptic exploration of the surfaces through the movement of their fingers and wrists.

#### Fine surface

A series of fine stimuli for Experiment 5 were produced with resin by a precision machine tool. The parameters of the global feature were the same as those of coarse surfaces (Fig. 1F), while the local feature was changed from a coarse surface (raised dot) to a finely textured surface by polishing the surface of resin stimuli (without any dots) with sandpaper with different levels of roughness (#80 or #240 mesh). This local feature, on the order of micrometers, was defined as a fine textural feature (Hollins and Bensmaïa 2007; Weber et al. 2013; Yoshioka et al. 2001). Ten unique haptic stimuli combining local features (polished by #80 or #240 mesh sandpaper) and global features (number of cycles: 0.5–2.5) were obtained.

### Experimental setup and perceptual task

**Fig. 2:**
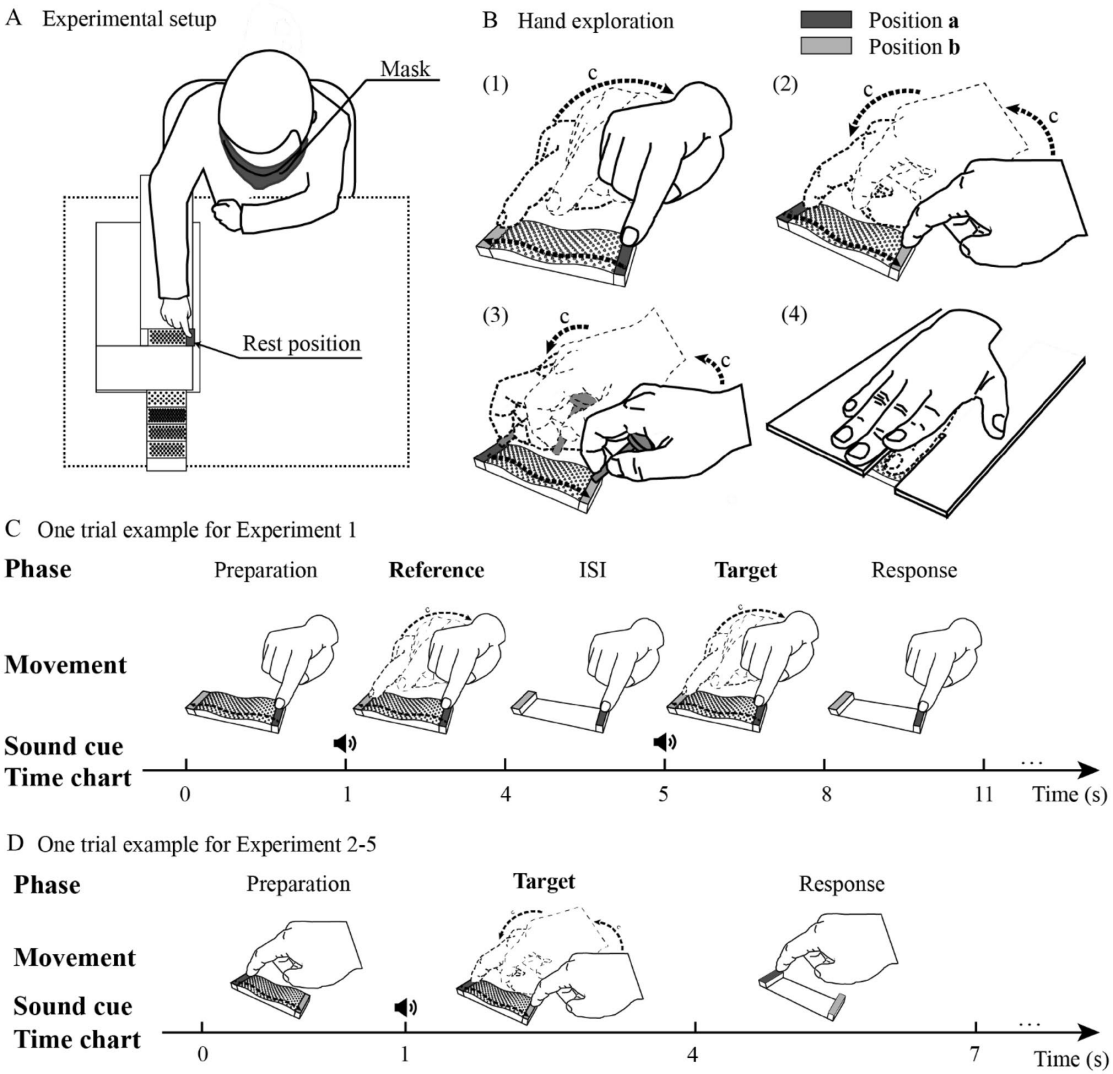
Experimental setup and procedure. **A** Participants’ position during the experiment**.** Stimuli were placed in a long container that could be slid along a track, and the subject's finger was always on the side of a fixed "touch window", under where the experimenter presented the stimulus to be explored. **B** Hand exploration movements in Experiments 1–4. Participants scanned the stimuli following the contour of the surface (from position “a” to position “b”) and back to the rest position “a” following trail “c”. **C** A single trial time chart of the task in Experiment 1. Participants first explored the reference stimulus and placed their finger on resting point "a" during the interstimulus interval (ISI) so that the experimenter could change the stimulus to the target stimulus by sliding the container over the track. The interval between the reference stimulus and the target stimulus was about 1 s. Both the reference stimulus and the target stimulus appeared in the same fixed location—below the "touch window. Participants then explored the target stimulus and placed their finger back on resting point "a" during the response phase. **D:** A single trial time chart of the task in Experiments 2–5 (Experiment 2 is shown as an example)

Each participant was seated comfortably with the tactile stimulator at approximately waist level (Fig. 2A). The participant’s right arm was comfortably supported on an independent manipulandum that allowed only elbow to rotate in the horizontal plane. The participant’s left arm was maintained in a comfortable resting position. Before the experiments began, the participant was blindfolded to prevent him or her from perceiving any visual information about the stimulus. During the intertrial interval, the distal phalanx of the right hand rested on the marker position for the next trial.

#### Magnitude estimation task

A transformation of the subjective magnitude estimation method was performed in Experiment 1. A stimulus with 1.5 cycles and 4 mm dot spacing was predefined as a reference stimulus. During the task, participants always scanned the reference stimulus before scanning the target stimuli.

A simple schematic of the task is provided in Fig. 2C. At the beginning of the trial, participants rested their fingers on the marker position “a”. After a sound cue, they started exploring the reference stimulus twice from left to right and back to the rest position (Fig. 2B). Then, the target stimulus sound cue was presented after a 1 s delay, and the participants were asked to explore the target stimuli. After finishing the exploration of both the reference and target stimuli, participants were asked to report the roughness magnitude of the target stimulus compared with that of the reference stimulus. Participants were informed at the beginning of the experiment that they needed to use a similar velocity and force for the exploration of each stimulus. The reference stimulus was defined as having a roughness magnitude of 50. Furthermore, participants were asked to indicate the roughness of the stimulus using a number from 1 to 100, with larger numbers representing rougher stimuli. During the experiment, no feedback was provided to the participants. Before the experiment, participants were allowed to perform 25 practice trials that included the exploration process to give the participants a general idea about the range of stimuli that would be presented.

To avoid the influence of the difference in familiarity caused by the reference stimulus, participants were asked to perform the magnitude estimation task without a reference stimulus in Experiments 2–5. A simple schematic of the procedure is outlined in Fig. 2D.

### Data and analysis

To avoid errors caused by the subjective scoring strategies of different participants, the z-score was calculated using the following equation:

1 $$Z_{i,r} = \frac{{O_{i} - M_{r} }}{{{\text{SD}}_{r} }}.$$

In Eq. 1, $i$ denotes the trial number and $r$ denotes the participant number. Thus, ${\mathrm{Z}}_{i,r}$ denotes the z-score of the $i$th trial of participant $r$. ${M}_{r}$ denotes the mean score of all trials of participant $r$. ${\mathrm{SD}}_{r}$ denotes the standard deviation of all trials of participant $r$. A repeated-measures analysis of variance (ANOVA) was performed on the normalized z-score. If Mauchly’s test of sphericity was violated, a Greenhouse–Geisser correction was applied. Post hoc tests were performed using paired-sample *t* tests with Bonferroni correction. The level of significance was fixed at *p* < 0.05 for all statistical analyses. All data processing and statistical analysis were performed in R (version 4.0.3) with the “bruceR” package.

### Experiment 1: The manner in which local and global features affect roughness estimation

#### Design

The experimental design comprised two within-subject variables: sinusoidal-like curves (number of cycles from 0.5 to 2.5) and dot spacing (from 2 to 6 mm). Each participant completed a total of 500 trials. Four sessions were conducted on 4 different days to avoid fatigue effects. For each session, a pseudorandom list of 125 trials was preestablished that interleaved number of cycles and dot spacing. Each of the 25 stimuli was presented 5 times in one session. Before the experiment, participants performed at least 25 practice trials that included exploration to give the participant a general idea about the range of stimuli to be presented.

#### Results

**Fig. 3:**
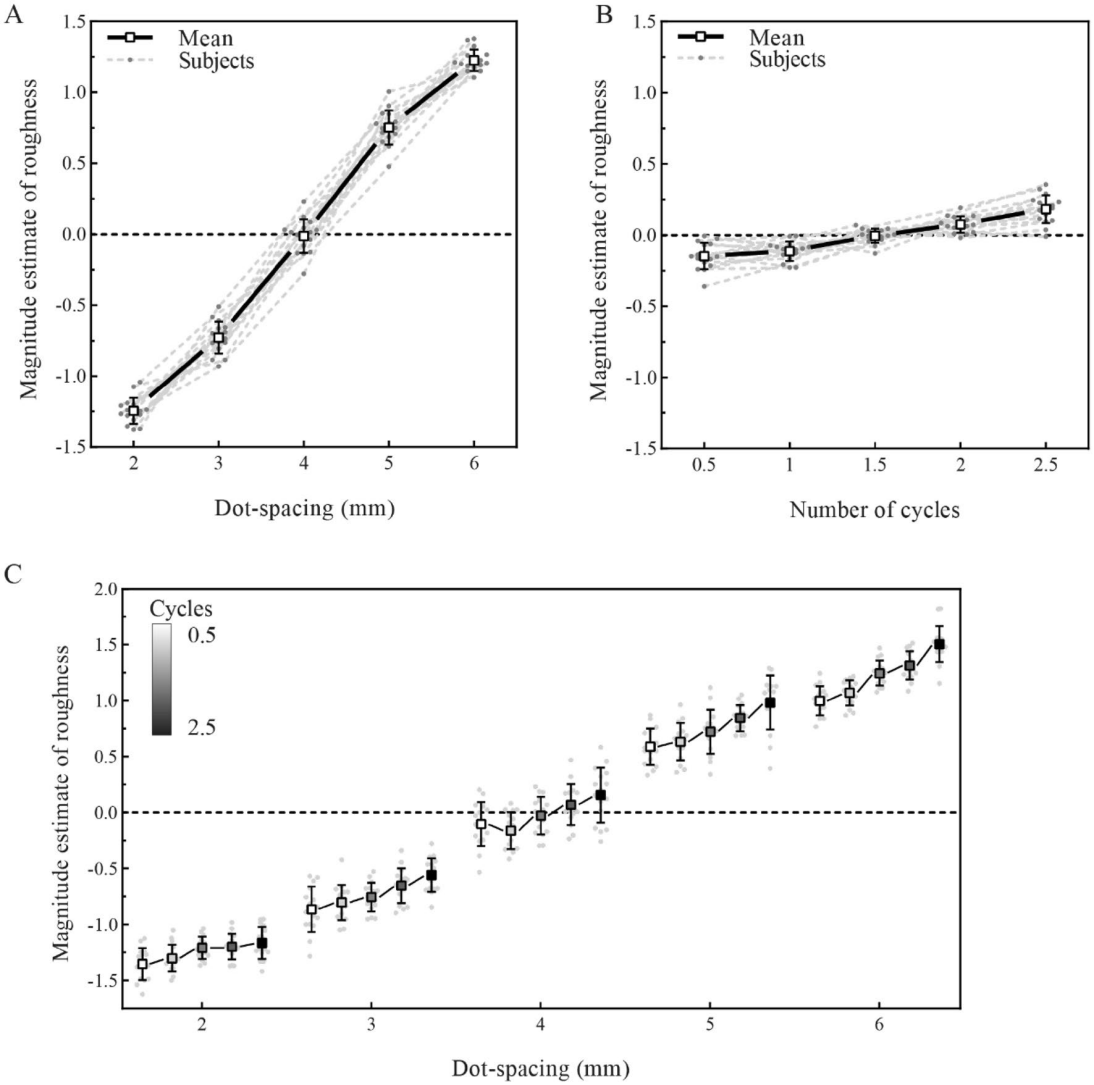
Effect of dot spacing and number of cycles on roughness estimation. **A** Individual and average function between the normalized magnitude estimation of roughness and dot spacing. Participants’ estimations of surface roughness showed a monotonic increase as the dot spacing increased. **B** Individual and average function between magnitude estimations of roughness and the number of cycles. Participants’ estimations of the roughness of the surface increased with an increase in the number of cycles. **C** A 5 × 5 plot for the effect of both dot spacing and number of cycles on roughness estimation. Compared with the global feature, local features had a greater influence on roughness estimation. Values are means ± SD

The individual data for all sixteen participants were plotted and are presented in Fig. 3. For dot spacing, an inspection of the individual curves indicated that roughness estimates showed a monotonic increase as dot spacing increased (Fig. 3A). For the number of cycles, although the trend was not as clear as that of dot spacing, the roughness estimates increased with an increasing number of cycles (Fig. 3B). Statistical analysis found significant main effects on the roughness estimation for both dot spacing (*F* (4, 28) = 1178, *p* < 0.001, *η*^2^*p* = 0.987) and the number of cycles (*F* (4, 28) = 40.7, *p* < 0.001, *η*^2^*p* = 0.732).

There was also a significant interaction (*F* (16, 240) = 3.40, *p* < 0.001, *η*^2^*p* = 0.185) in Experiment 1. The simple effects indicated that the number of cycles affected the roughness estimation on each level of dot spacing significantly, but when dot spacing reached 5 and 6 mm, the effect size of the number of cycles became larger (dot spacing − 2 mm: *F* (4, 15) = 6.58, *p* = 0.003, *η*^2^*p* = 0.637, dot spacing − 3 mm: *F* (4, 15) = 6.63, *p* = 0.003, *η*^2^*p* = 0.639, dot spacing − 4 mm: *F* (4, 15) = 5.78, *p* = 0.005, *η*^2^*p* = 0.607, dot spacing − 5 mm: *F* (4, 15) = 19.3, *p* < 0.001, ***η***^**2**^***p = 0.838***, dot spacing − 6 mm: *F* (4, 15) = 32.3, *p* < 0.001, ***η***^**2**^***p = 0.896***).

The post hoc comparison was adjusted by the Bonferroni method for 10 tests at each dot spacing level. The results showed that the roughness ratings increased overall with an increasing number of cycles. Moreover, just as the simple effect showed that number of cycles had a greater effect on roughness estimation when dot spacing was large (e.g., when dot spacing was 6 mm, number of cycles 2.5 vs. 2: *t* (15) = 4.49, *p* = 0.0004, Cohen's *d* = 1, number of cycles 2.5 vs. 1.5: *t* (15) = 5.83, *p* < 0.0001, Cohen's *d* = 1.36, number of cycles 2.5 vs. 1: *t* (15) = 9, *p* < 0.0001, Cohen's* d* = 2.28, number of cycles 2.5 vs. 0.5: *t* (15) = 9.75, *p* < 0.0001, Cohen's *d* = 2.66; when dot spacing was 2 mm, number of cycles 2.5 vs. 2: *t* (15) = 2.43, *p* = 0.028, Cohen's *d* = 0.18, number of cycles 2.5 vs. 1.5: *t* (15) = 1.24, *p* = 1, Cohen's *d* = 0.23, number of cycles 2.5 vs. 1: *t* (15) = 3.38, *p* = 0.0041, *Cohen's d* = 0.72, number of cycles 2.5 vs. 0.5: *t* (15) = 4.77, *p* = 0.0002, Cohen's *d* = 1).

#### Discussion

Experiment 1 demonstrated that roughness estimation was significantly affected by local features (dot spacing). This finding is consistent with numerous studies, which showed that the roughness estimation of raised-dot surfaces monotonically increased as dot spacing increased (Dépeault et al. 2009; Goodman and Bensmaia 2017; Sutu et al. 2013). Beyond these previous studies, the global feature (number of cycles) of the stimuli also affected roughness estimations. Specifically, people felt that a raised-dot surface with more curves was rougher than one with fewer curves (Fig. 3B).

However, the curve changes also resulted in a change in the contact area between the participants’ fingers and the stimulus. For instance, as the curvature of a single curve increased, the surfaces changed more drastically. During the exploration in Experiment 1, the contact between the participants’ skin and stimulus became larger when the stimuli had more curves. This change was exacerbated by finger movement (e.g., rotation of the finger during exploration). To avoid this confounder, we changed the direction of finger movement from lateral movement to forward and backward movement in Experiment 2, which allowed the participants to more easily complete contour-following exploration, avoided unnecessary finger rotation and reduced the difference in the contact area while changing the number of cycles.

### Experiment 2: Interaction of local and global features on roughness estimation

#### Design

In Experiment 2, to observe a clear interaction of local and global features on roughness estimation, only stimuli with 0.5 and 2.5 cycles were selected from the stimuli used in Experiment 1. Thus, a total of 10 stimuli in Experiment 2 were used (2 levels of cycles; 5 levels of dot spacing). Each participant completed a total of 200 trials that were divided into 4 blocks. For each block, a pseudorandom list of 50 trials was preestablished that interleaved curve and dot spacing. Each of the 10 stimuli was presented 5 times in one block. A 5-min rest period occurred between blocks to avoid fatigue. Before the experiment, participants completed at least 10 practice trials that included exploration so that they could have a general idea about the range of stimuli to be presented.

#### Results

**Fig. 4:**
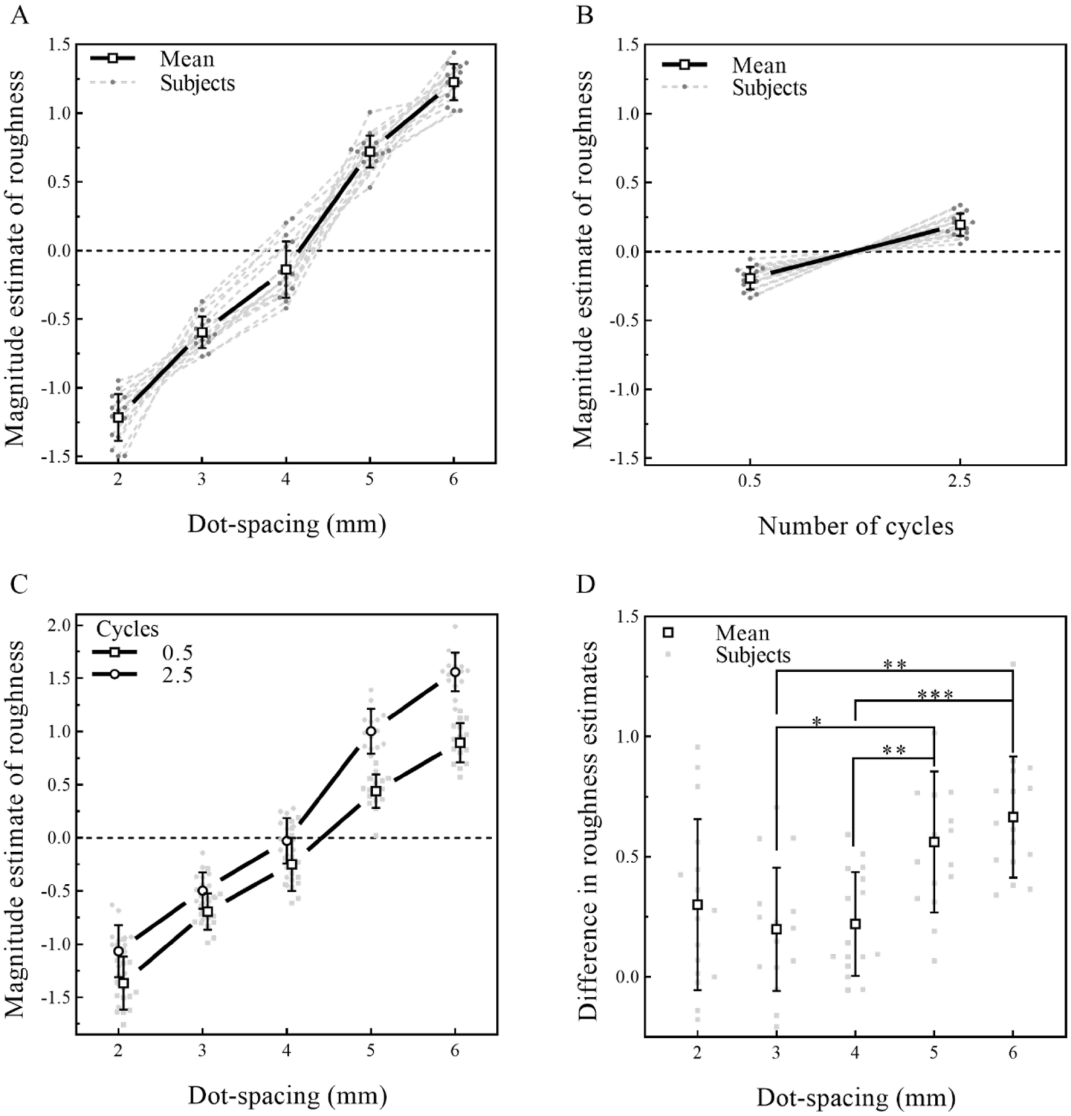
Effect of dot spacing and number of cycles on roughness estimation. **A** Individual and average function between normalized magnitude estimations of roughness and dot spacing. The result is the same as that in Experiment 1, in which participants’ estimations of the roughness of the surface increased with increasing dot spacing. **B** Individual and average scatter plots of magnitude estimations of roughness and the number of cycles. Participants’ estimations of the roughness of the surface with more cycles of curves were rougher. **C** Interaction between the number of cycles and dot spacing on roughness estimation. **D** To better show the interaction between the number of cycles and dot spacing on roughness estimation, the individual and the average difference in the magnitude estimations of roughness between stimuli with 0.5 and 2.5 cycles in each level of dot spacing was plotted. Values are the means ± SD. *P < 0.05, **P < 0.01, and ***P < 0.001

The individual data for all sixteen participants were plotted and are presented in Fig. 4. For dot spacing (Fig. 4A), an inspection of the individual curves indicated that roughness estimates monotonically increased as dot spacing increased. For the number of cycles (Fig. 4B), the roughness estimates of stimuli with more cycles were larger. Statistical analysis showed that both dot spacing (*F* (1.56, 23.3) = 531, *p* < 0.001, *η*^2^*p* = 0.973) and the number of cycles (*F* (1, 15) = 88.5, *p* < 0.001, *η*^2^*p* = 0.855) had a significant main effect.

The interaction between dot spacing and the number of cycles was also significant (*F* (1.77, 26.4) = 11.3, *p* < 0.001, *η*^2^*p* = 0.43) (Fig. 4C). The post hoc comparison (corrected by the Bonferroni method for 10 tests) indicated that curves had a significant effect on the estimation of roughness at each dot-spacing level. To better show the interaction between the curves and dot spacing on roughness estimations, for every participant, we calculated the difference in the magnitude estimation of roughness among stimuli with different global features at each level of dot spacing (Fig. 4D). The result showed that compared with the stimuli with dot spacing of 3 and 4 mm, the effect of the global feature on the magnitude estimation of roughness for the stimuli with dot spacing of 5 and 6 mm was significantly increased (dot spacing 5 mm vs. 3 mm: *t* (15) = 3.9, *p* = 0.0014, Cohen's *d* = 0.96; dot spacing 5 mm vs. 4 mm: *t* (15) = 4.95, *p* = 0.0002, Cohen's *d* = 0.90; dot spacing 6 mm vs. 3 mm: *t* (15) = 4.34, *p* = 0.0006, Cohen's *d* = 1.23; dot spacing 6 mm vs. 4 mm: *t* (15) = 6.94, *p* < 0.0001, Cohen's *d* = 1.18).

#### Discussion

In Experiment 2, we changed the finger movement direction from a horizontal to a vertical orientation to reduce the effect of finger rotation. The change in hand motion did not influence our previous finding in Experiment 1 (Fig. 4A, B). One possible explanation for this outcome is that cutaneous input provides information about both local and global features (Hsiao 2008), which is coded by cutaneous afferents (Goodman and Bensmaia 2017; Hollins and Bensmaïa 2007; Jenmalm et al. 2003; Saal and Bensmaia 2014; Yau et al. 2016). Thus, the global feature affects the coding of the spatial information regarding the roughness of the material, thereby affecting participants’ roughness estimations. Both local and global features had a stable effect on roughness estimations with motion invariance, suggesting that the variance in roughness estimation caused by global features did not come from hand motion. While hand motion is important for us to accurately extract roughness information or even form constant perceptual images of roughness (Boundy-Singer et al. 2017; Meftah et al. 2000; Saal et al. 2018; Yoshioka et al. 2011), only changes in the movement did not modulate the influence of each physical feature on roughness estimation.

However, although spatial coding dominates the perception of the roughness of coarse surfaces (macrostructure) (Hollins and Bensmaïa 2007; Hollins and Risner 2000; Hollins and Bensmaïa 2007; Gescheider and Wright 2013), it is difficult to know whether the effect of global features on roughness estimation relies exclusively on encoding from spatial information. Temporal information (texture-specific vibration) also plays an important role in roughness perception, especially for fine surfaces (microstructure) (Fagiani and Barbieri 2016; Weber et al. 2013). In fact, even for the roughness estimation of coarse raised-dot surfaces, the vibration (temporal information) of the skin caused by the texture of the surface is still very important information (Weber et al. 2013). Therefore, for a deeper understanding of the mechanism of roughness estimation, we examined this question in Experiment 3.

### Experiment 3: Local and global features affect roughness estimation during indirect exploration

#### Design

The experimental design and procedure were identical to those in Experiment 2 except for the exploration method. More specifically, participants were instructed to place the distal phalanx of their index fingers against the front end of the probe (Fig. 1D) and their palm against the base of the probe; then, they were to use their remaining fingers to control the probe without sliding sideways (Fig. 2B). Before the formal experiment, participants completed at least 15 practice trials that included exploration using the probe so that they could have a general idea about the range of stimuli that would be presented.

To avoid the effect of sound on roughness, the sound was blocked in Experiment 3 with earmuffs and white noise. White noise was played through in-ear headphones, and acoustic earmuffs (3M Peltor-X4A) were added externally to completely block the sound generated by exploration during the experiment. The volume of the white noise was adaptive to individuals. Before starting the experiment, the experimenter slid the probe over stimuli and asked the participant to raise his or her hands if he or she could hear the sound. Next, the experimenter adjusted the volume of the white noise until the participant had no response to the sliding of the probe, which signified that the participant could no longer hear the sound caused by the probe. Finally, the participant completed the exploration on his or her own and confirmed that he or she could not hear the sound.

#### Results

**Fig. 5:**
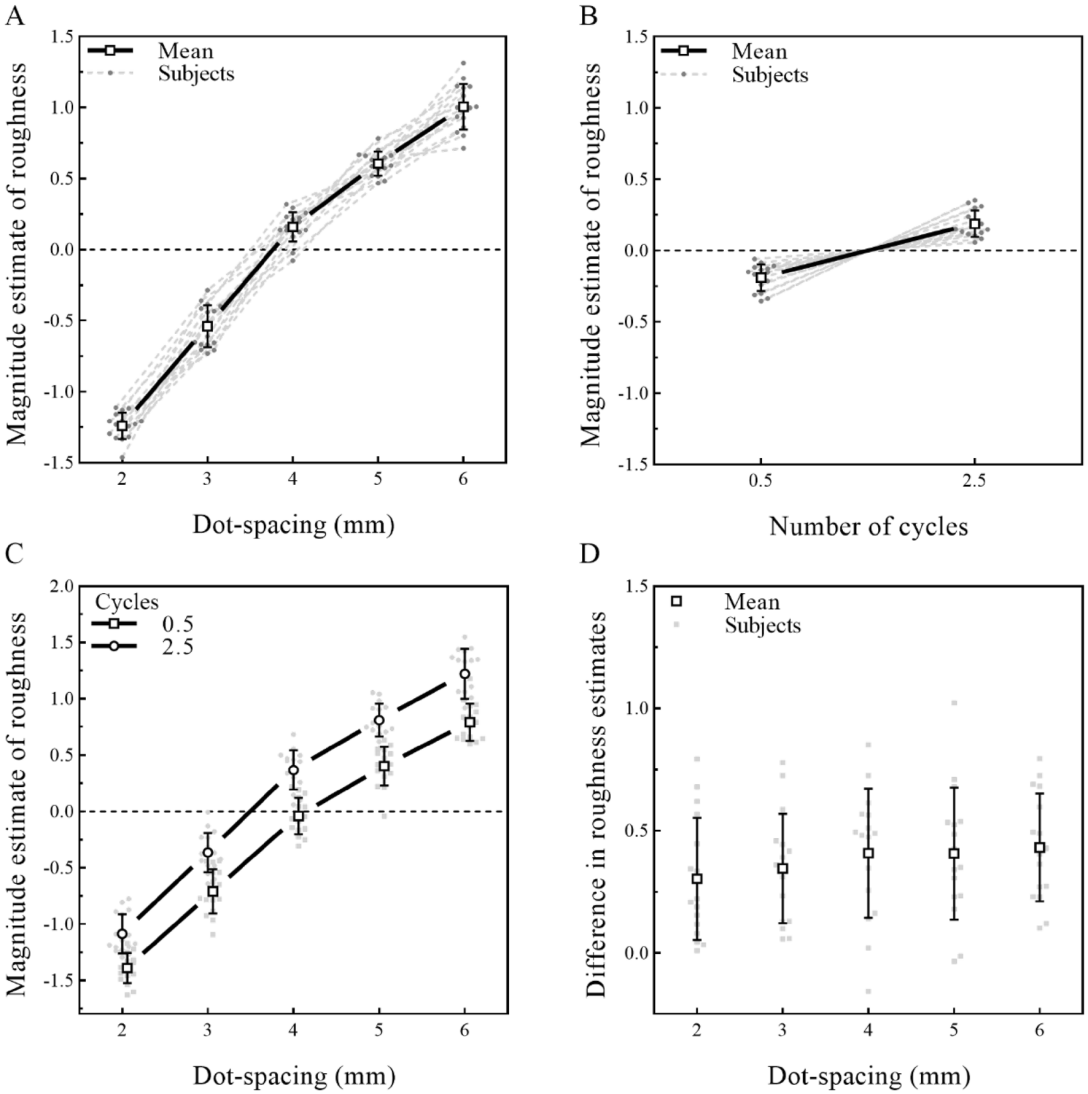
Effect of dot spacing and the number of cycles on roughness estimation. **A** Individual and average function between normalized magnitude estimations of roughness and dot spacing. Participants’ estimations of the roughness of the surface showed a monotonic increase as the dot spacing increased. **B** Individual and average scatter plots of magnitude estimations of roughness and the number of cycles. Participants’ estimations of the roughness of the surface increased with an increase in the number of cycles. **C** There is no significant interaction between number of cycles and dot spacing on roughness estimation. **D** Individual and average scatter plots of the difference in the magnitude estimations of roughness between stimuli with 0.5 and 2.5 cycles of curves in each level of dot spacing. There was no significant difference among the estimations. Values are the means ± SD

The individual data for all sixteen participants, which were similar to those of Experiments 1 and 2, were plotted and are presented in Fig. 5. We found significant main effects on roughness estimation for both dot spacing (*F* (2.48, 37.2) = 690, *p* < 0.001, *η*^2^*p* = 0.979) and the number of cycles (*F* (1, 15) = 32.14, *p* < 0.001, *η*^2^*p* = 0.814). Moreover, as shown in Fig. 5C, D, there was no significant interaction in Experiment 3 (*F* (1, 15) = 1.4, *p* = 0.253, *η*^2^*p* = 0.086), which is different from the outcomes observed in Experiments 1 and 2.

#### Discussion

In Experiment 3, we found that even though participants used indirect exploration with a rigid probe, the local feature still affected the roughness estimation, which was similar to the outcome observed in Experiment 2. This result was consistent with previous studies showing that humans can not only perceive roughness through direct skin contact but also perceive roughness effectively through indirect contact with tools (Klatzky et al. 2003; Yoshioka et al. 2011). Furthermore, as we expected, we found a significant difference in roughness estimation between the stimuli with 0.5 cycles of curves and 2.5 cycles of curves. The exploration method did not change the main effect of the global feature on roughness estimation. Thus, for coarse surfaces (raised-dot surfaces), the global feature stably affected the roughness estimation, whether through spatial or temporal encoding.

However, due to the curved surface, the dot spacing of the convex area was slightly greater than the dot spacing of the convex area. We believe that this small change in dot spacing was not sufficient to explain the influence on roughness estimation; however, it still led to a possible explanation for our result that the effect of the global feature on roughness estimation was derived from the change in local input caused by the global feature, which may lead to a salience-driven overestimation phenomenon. According to the idea of peak bias (Cataldo et al. 2019; Walsh et al. 2016), if the global feature leads to a different perception of local information of the different areas of the stimulus (convex and concave), the salience of the rougher area has a large influence on the roughness perception as a whole and lead to an overestimation of the roughness of the whole stimulus. Moreover, in Experiments 1–3, we used raised-dot surfaces as stimuli. It was difficult to show whether the global feature itself would affect the roughness estimation. More specifically, when there is no raised-dot texture on the surface of the object, how does the global feature affect the roughness judgment? Experiments 4 and 5 were designed to investigate these two matters.

### Experiment 4: Effect of local and global features on roughness estimation during static exploration

#### Design

In Experiment 4, only stimuli with 1, 1.5, and 2.5 cycles of curves and with dot spacings of 2, 4, and 6 mm were selected from the stimuli used in Experiment 1. Thus, a total of 9 stimuli in Experiment 4 were used (3 levels of cycles; 3 levels of dot spacing). The experimental design comprised three within-participant variables: curvature (radius of curves: 19.6, 55.1, 124.8 mm), area of the curve (convex or concave), and dot spacing (2, 4, 6 mm). Each participant completed a total of 180 trials. Two blocks took place separated by a 5-min rest period to prevent participant fatigue. For each block, a pseudorandom list of 90 trials was preestablished that included the interleaved curve, curvature, and dot spacing. The experimental procedure was identical to that in Experiment 2, except participants could only press their index fingers up and down rather than sliding on the stimuli (Fig. 2B). Before the experiment, participants completed at least 18 practice trials that included exploration so that they could have a general idea about the range of stimuli to be presented.

#### Results

**Fig. 6:**
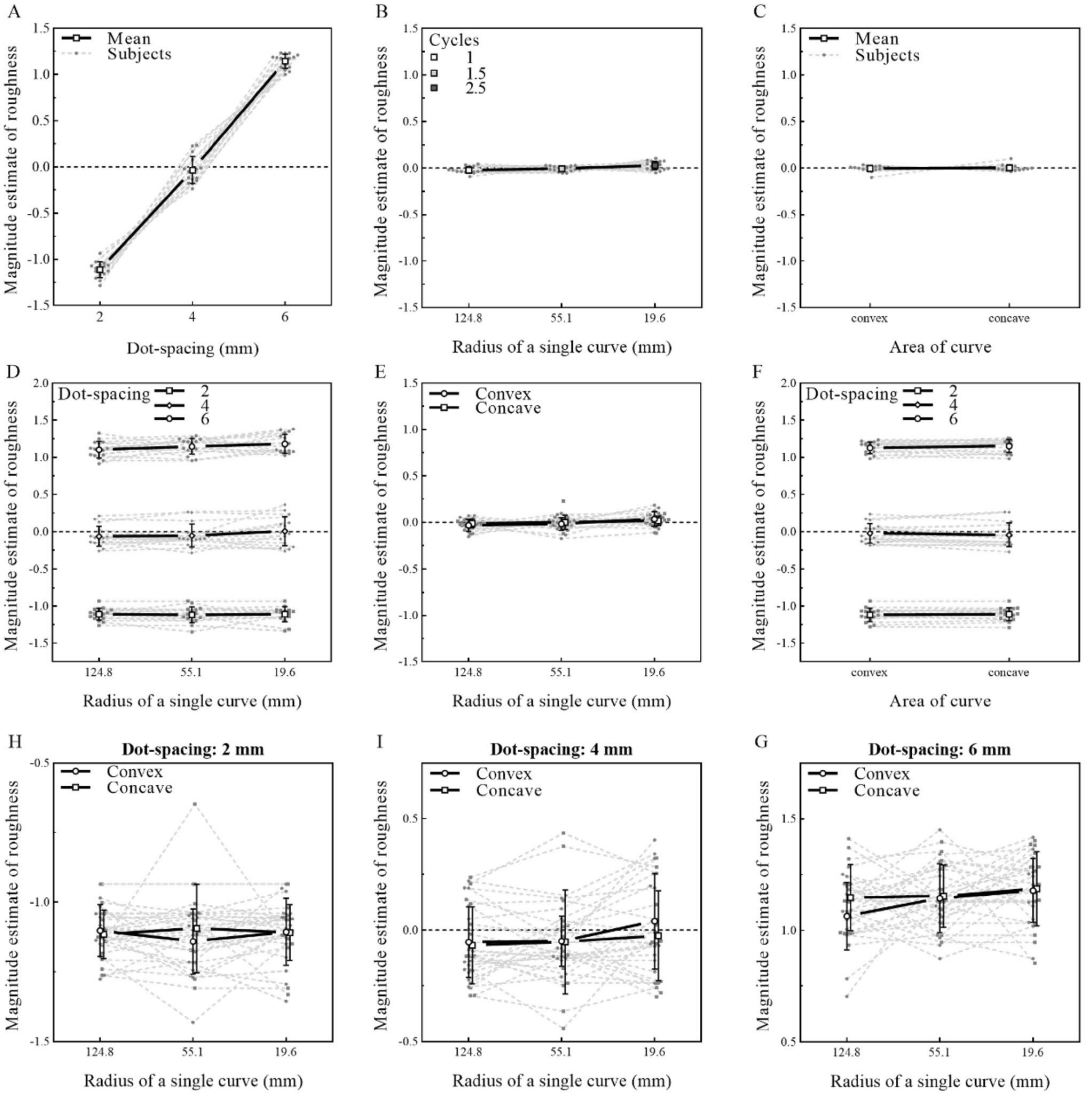
Effect of dot spacing and the number of cycles on roughness estimation during static exploration. **A** Individual and average function between normalized magnitude estimations of roughness and dot spacing. The result is the same as those in Experiments 1, 2 and 3, in which participants’ estimations of the roughness of the surface increased with increasing dot spacing. **B** Individual and average function between normalized magnitude estimations of roughness and the radius of the curves. The roughness rating shows a tendency to increase as the radius of a single curve decreases. The label shows the number of cycles corresponding to the radius of a single curve. **C** Individual and average results of normalized magnitude estimations of roughness and curvature. There was no significant difference between participants’ roughness estimations of convex and concave areas. **D** No significant interaction between dot spacing and the radius of a single curve. **E** No significant interaction between dot spacing and curvature. **F** No significant interaction between curvature and the radius of a single curve. **H**–**G** No significant high-order interaction among dot spacing, radius of the curve, and area of the curve. Values are the means ± SD

The individual data for all sixteen participants were plotted and are presented in Fig. 6. A three (dot spacing: 2, 4, 6 mm) × three (number of cycles: 2, 3, 5) × two (curvature: convex or concave) repeated-measures ANOVA was performed on the normalized z-score. Only dot spacing (*F* (1.16, 11.3) = 1191, *p* < 0.001, *η*^2^*p* = 0.988) and the radius of the curves (*F* (1.47, 22.03) = 4.85, *p* = 0.026, *η*^2^*p* = 0.244) had a significant main effect, while there was no significant difference between the roughness estimation of the convex and concave areas of the stimuli (*F* (1, 15) = 0.12, *p* = 0.732, *η*^2^*p* = 0.008). Furthermore, there was no significant interaction in Experiment 4 (dot spacing and radius of the curve: *F* (2.66, 39.9) = 0.96, *p* = 0.412, *η*^2^*p* = 0.06; dot spacing and area of the curve: *F* (1.15, 17.2) = 1.83, *p* = 0.194, *η*^2^*p* = 0.109; area of the curve and radius of the curve: *F* (1.7, 25.5) = 0.6, *p* = 0.531, *η*^2^*p* = 0.038; dot spacing, radius of the curve and area of the curve: *F* (3.12, 46.9) = 0.89, *p* = 0.455, *η*^2^*p* = 0.056).

#### Discussion

In Experiment 4, we found that even in the state of static touch, there was a strong effect of dot spacing on roughness estimation. Consistent with previous studies, for coarse surfaces, the local feature could be spatially encoded and had a strong influence on roughness estimation during static touch (Hollins and Risner 2000; Saal and Bensmaia 2014; Weber et al. 2013). There was a weak effect of the radius of a single curve on roughness estimation (Fig. 6B). This may have occurred, because the global feature varied on the order of centimeters, which makes it difficult to be perceived by only cutaneous information through static touch. Thus, although not necessary, movement plays an important role in the influence of global features on roughness estimation, which was consistent with the outcomes of Experiment 3 and shows the importance of temporal encoding. Furthermore, there was no significant difference between roughness estimation of the convex and concave areas of stimuli. This result indicated that the faint physical difference in dot spacing between convex and concave areas did not lead to a significant change in roughness estimation. Therefore, we suggest that the change in local input caused by the global feature (salience-driven overestimation) was not the main reason for the influence of global features on roughness estimation.

### Experiment 5: Interaction of local and global features on fine surface roughness estimation

#### Design

The design and procedure were identical to those used in Experiment 2 except that only fine surfaces were used as stimuli in Experiment 5.

#### Results

**Fig. 7:**
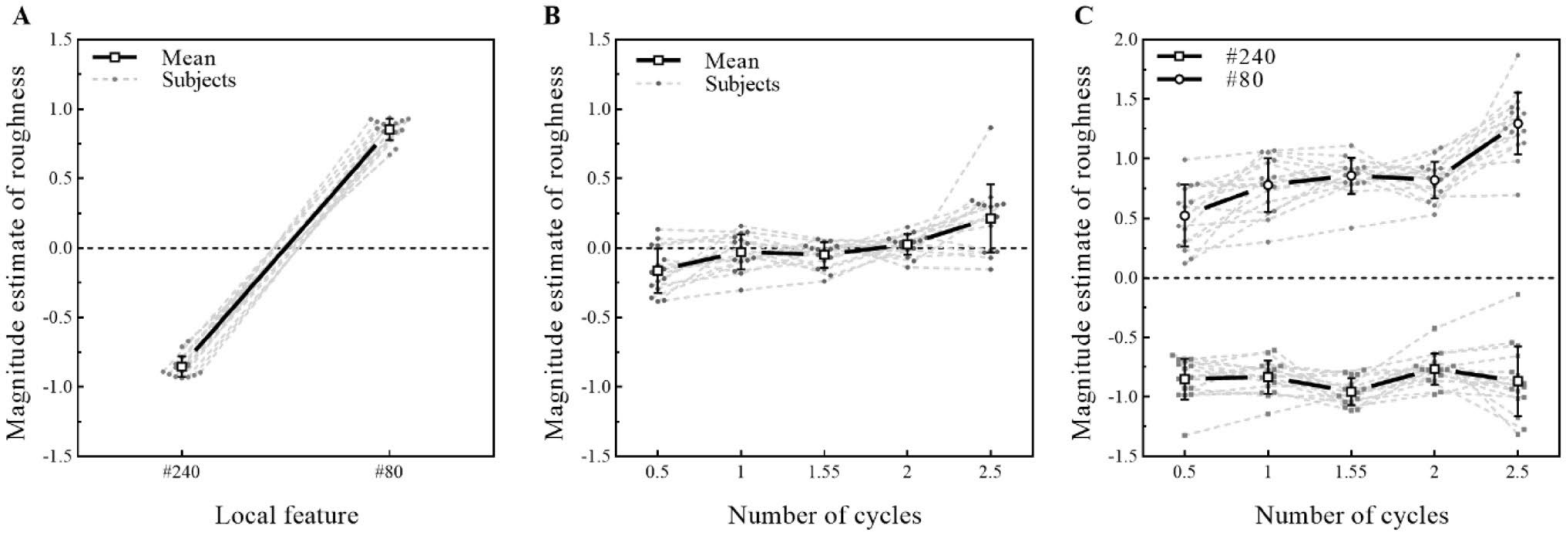
Effect of local features and the number of cycles on roughness estimation. **A** Individual and average scatter plots of magnitude estimations of roughness and local features. People felt that the surface polished with #80 mesh sandpaper was rougher than the surface polished with #240 mesh sandpaper. **B** Individual and average functions between normalized magnitude estimations of roughness and global features. The tendency of roughness estimation with the number of cycles was very similar to that in Experiment 1, but there was no significant difference in the roughness estimation of stimuli with 1–2 cycles of curves. **C** The effect of the global feature on roughness estimation was modulated by local features. The number of cycles showed a strong effect on roughness estimation when the stimuli polished with #80 mesh sandpaper were presented. For these stimuli polished with #240 mesh sandpaper, the number of cycles showed a weak effect on roughness estimation. Values are the means ± SD

The individual data for all 16 participants were plotted and are presented in Fig. 7. A five (number of cycles) × two (local feature) repeated-measures ANOVA was performed on the normalized z-scores. Figure 7A shows that there was a significant main effect of local features (*F* (1, 15) = 1970, *p* < 0.001, *η*^2^*p* = 0.992) on the roughness estimation, which indicated that local features that changed by polishing stimuli with sandpapers of different meshes significantly affected the roughness estimation of stimuli. People felt that the surface polished with #80 mesh sandpaper was rougher than the surface polished with #240 mesh sandpaper. Figure 7B shows that the number of cycles (*F* (1.58, 23.7) = 10.55, *p* = 0.001, *η*^2^*p* = 0.413) also had a significant main effect. The tendency was partly similar to the results of Experiment 1 except that there was no significant difference in the roughness estimation of stimuli with 1–2 cycles of curves.

Furthermore, the interaction between local features and the number of cycles was significant (*F* (2.21, 33.08) = 26.3, *p* < 0.001, *η*^2^*p* = 0.63) (Fig. 7). The post hoc comparison (corrected by the Bonferroni method for 10 tests) indicated that global features had a significant effect on the estimation of roughness for the stimuli polished with #80 mesh sandpaper. However, when the stimuli were polished with #240 mesh sandpaper, the influence of global features on roughness estimation almost disappeared. More specifically, for stimuli polished with #80 mesh sandpaper, although stimuli with 1–2 cycles of curves were considered to result in no significant difference in roughness estimation, stimuli with large cycle curves were perceived to be significantly smoother than other stimuli (e.g., number of cycles 0.5 vs. 1: *t* (15) = 4.21, *p* = 0.0008, Cohen's *d* = 0.67; number of cycles 0.5 vs. 1.5: *t* (15) = 5.78, *p* < 0.0001, Cohen's *d* = 0.87; number of cycles 0.5 vs. 2: *t* (15) = 4.08, *p* = 0.001, Cohen's *d* = 0.77; number of cycles 0.5 vs. 2.5: *t* (15) = 6.36, *p* < 0.0001, Cohen's *d* = 2.02), and stimuli with small cycle curves were perceived to be significantly rougher than other stimuli (e.g., number of cycles 2.5 vs. 1: *t* (15) = 4.7, *p* = 0.0003, Cohen's *d* = 1.35; number of cycles 2.5 vs. 1.5: *t* (15) = 4.48, *p* = 0.0004, Cohen's *d* = 1.35; number of cycles 2.5 vs. 2: *t* (15) = 6.12, *p* < 0.0001, Cohen's *d* = 1.24). For these stimuli polished with #240 mesh sandpaper, there was no significant difference among the roughness estimation of stimuli with different curves (except that number of cycles 2 vs. 1.5: *t* (15) = 5.69, *p* < 0.0001, Cohen's *d* = 0.5).

#### Discussion

The results showed that participants could perceive the roughness of fine surfaces effectively through temporal information by sliding their fingers along these surfaces (Hollins and Bensmaïa 2007; Natsume et al. 2019; Weber et al. 2013; Yoshioka et al. 2001). Global feature still has a significant effect on roughness estimation. It also provide evidence to support that the faint physical difference in dot spacing between convex and concave areas is not the mean factor contributing to the global feature affecting roughness estimation. However, the interaction showed that the influence of global features on roughness estimation, such as in Experiment 1, only occurred on the stimuli polished with #80 mesh sandpaper. For the smoother stimuli that were polished with #240 mesh sandpaper, the global feature had little effect on roughness estimation. This result indicated that the influence of global features on roughness estimation was modulated by local features. In addition, when the size of local features was small to a certain extent, the influence of the global feature on the roughness estimation almost disappeared. Therefore, we inferred that the global feature itself did not affect the roughness estimation, but the interaction between the global and local features affected the roughness estimation.

## General discussion

The present study showed that human roughness estimation is affected by local features regardless of whether the surface is coarse or fine. In addition, there is a monotonic relationship between roughness estimation and dot spacing rather than the inverted U-shaped psychophysical curve that was found in a series of classical experiments (e.g., Connor et al. 1990; Blake et al. 1997). However, the range of dot spacing used in the present study was similar to that used in the previous study. The height of the raised dots could reasonably explain the discrepancy between the present study and Connor’s study (Sutu et al. 2013). For the surfaces with high dots (1.5 mm in our study and 1.8 mm in Sutu’s study), an individual’s roughness estimation monotonically increased as the dot spacing increased over 2–6 mm, while for the surfaces with low dots (0.35 mm in Connor’s study, lower than 0.62 mm in Blake’s study, and 0.36 mm in Sutu’s study), an inverted U-shaped psychophysical curve that peaked at 3 mm was found. Interestingly, roughness estimation is modulated by both dot spacing and dot height and is determined by the deformation of the skin, thus explaining the aforementioned phenomenon. We suggest that the function between dot spacing and roughness is still an inverted U-shaped curve, but the increase in height of dots delays the position of the peak to more than 6 mm in the present study.

The global feature likewise exhibits a stable influence on the roughness estimation during direct touch. The result emphasizes the crucial role of cutaneous afferents in processing macroscopic roughness information that integrates multiple pieces of information during direct exploration. Pertinently, cutaneous afferents are sensitive to the deformation of our skin during exploration; this skin deformation provides spatial information that is important for the coding of local texture (Drewing 2016; Goodman and Bensmaia 2017; Hollins and Bensmaïa 2007; Lieber et al. 2017; Saal and Bensmaia 2014; Weber et al. 2013) and global features (Goodwin et al. 1995, 1997; Goodwin and Wheat 2004; Sathian 2016; Yau et al. 2016). Therefore, we suggest that when the skin is in direct contact with an object, the changes in global features and local texture jointly change the deformation of the skin and further affect the perception of roughness.

More interestingly, the effect of global features on roughness was still significant even when the form of roughness changed (from spatial to temporal). Cutaneous input about spatial information is invalid for the perception of both local and global features of stimuli during indirect touch. People can perceive vibrations caused by local features (Klatzky et al. 2003; Lawrence et al. 2007; Yoshioka et al. 2011) and the kinesthetic input for the global feature. In this case, people perceive roughness through temporal information (Cascio and Sathian 2001; Hollins and Bensmaïa 2007; Lieber et al. 2017; Meftah et al. 2000). The present study demonstrated that roughness perception is still affected by global features during indirect touch. This result suggested that the influence of global features on roughness estimation depends not only on the spatial information of roughness encoded by cutaneous afferents but also on the temporal coding of indirect touch or finely textured surfaces. It also emphasizes the importance of kinesthetic, proprioceptive, and temporal information on roughness estimation. One possible explanation for this result is that the perception of global and local features did not occur through completely separate pathways (Stilla and Sathian, 2008). Information about different properties may be integrated into the brain (Kim et al. 2015; Sathian 2016; Yang et al., 2021a, b). Our recent study also provided evidence to support this idea by demonstrating that haptic curve processing and roughness processing share a large proportion of cortical networks (Yang et al. 2021a, b). Moreover, information processing of different properties may share the same system and, therefore, interact. For example, there may be a generalized system for processing magnitudes of different features that lead to mutual influences among different features during magnitude estimation (Bueti and Walsh 2009; Dormal and Presenti 2012; Yates et al. 2012).

There was a significant interaction between local and global features during direct touch. One possible explanation is that type 1 SAs dominate the coding for roughness perception of coarse surfaces (Hollins and Bensmaïa 2007; Weber et al. 2013), which makes the degree of skin deformation very important for roughness perception (Drewing 2016; Lederman 1974; Weber et al. 2013). Large dot spacing caused a greater intrusion of the participants’ fingers into the interval between the elements; hence, the skin deformation caused by the curve change also became larger, which led to an increase in the magnitude estimations of roughness.

Moreover, the interaction between local and global features that existed in direct touch (Experiments 1, 2) was not evident in indirect touch (Experiment 3); this outcome showed that direct contact between the skin and the surface played an important role in how local features moderate the effect of the global feature on roughness estimation. We suggest that the direct surface contact produced a spatial map of activation in type 1 SAs, which directly coded spatial roughness information. When participants held probes, the roughness was only coded temporally by vibrations through the probe (Hollins and Bensmaïa 2007; Johnson et al. 2002; Klatzky et al. 2003; Lawrence et al. 2007; Weber et al. 2013). This indirect encoding caused the participants to lose part of the spatial information, which is very important for roughness estimation of a coarse surface. Therefore, the change in the exploration method decreased the interaction between local and global features in Experiment 3.

A series of finely textured surfaces were also used as stimuli in the present study and led to similar but not identical results to the experiments in which coarse surfaces were used as stimuli. Compared with a coarse surface, a fine surface reduced the influence of global features on the roughness perception. Thus, although we did not distinguish the similarities between the temporal encoding that occurred in Experiment 3 and Experiment 5, the temporal encoding of the raised-dot surface by indirect exploration and the temporal encoding of a finely textured surface by direct exploration may not be the same. Especially in the state of direct exploration, for coarse surfaces, roughness perception was highly dependent on spatial information, while for fine surfaces, roughness perception was highly dependent on temporal information (Goodman and Bensmaia 2017; Hollins and Bensmaïa 2007; Meftah et al. 2000; Weber et al. 2013). A possible explanation for this result is that the temporal information received by the indirect exploration of coarse surfaces was not directly used in the roughness estimation but was transformed into spatial information and then took part in the spatial encoding for roughness perception. For fine surfaces, the vibration generated by fingerprints was directly used as the temporal encoding for roughness. Therefore, this difference in encoding format may have caused changes in the interaction between the global and local features in the perception process, which led to changes in the weight of global information on roughness perception. Collectively, our findings provided behavioral evidence to support the view that the information of both local texture and global features contribute to roughness perception (Isett et al. 2018).

Another possible interpretation of our findings is that roughness is a high-level representation. In the peripheral nervous system, the variation in the population response of a different type of fiber accounts for roughness perception; more specifically, type 1 SAs are sensitive to spatial information about coarse surfaces detected by the skin detected (spatial density of raised-dot surfaces), and RA receptors and PC are sensitive to temporal information about fine surfaces detected by the skin (vibration during the touch of a fine surface) (Connor et al. 1990; Hollins and Bensmaïa 2007; Gescheider et al. 2010; Gescheider and Wright 2013; Weber et al. 2013; Liber et al. 2017). These two types of information are integrated (but nonlinear) in the somatosensory cortex and form a constant perception of roughness (Lieber and Bensmaia 2019,2020). However, beyond the early sensory areas, it seems that the formation of roughness perception is more dependent on the activation of the association area cortex (Eck et al. 2016). This is consistent with our results that people form roughness perceptions that not only depend on a single physical feature but also the processing of features from different dimensions that are then all combined into one roughness perception that requires the involvement of more association area cortices.

Moreover, top-down modulation during roughness estimation may also explain our result. First, active motion seems to have a very important contribution to the accuracy of our roughness judgments, and our top-down encoding of movements is likely to be an important factor in our maintenance of a stable roughness perception (Yoshioka et al. 2011). This means that the human sensory system is typically considered to have a bidirectional hierarchy rather than a strictly bottom-up hierarchy (Lange et al. 2018; Kanai et al. 2015; Park and Friston 2013). Processing along the bottom-up feedforward pathway is thought to produce increasingly complex internal representations of the sensory input feature-by-feature, whereas processing that follows the top-down feedback pathway is thought to enhance the representation of sensory information on the basis of prior experiences. For example, our previous study (Yu et al. 2019) demonstrated that the human primary somatosensory cortex received top-down predictive feedback during predictions based on information detected by the participants’ fingers. Furthermore, information from other sensory modalities may contribute to haptic roughness estimation (Yang, et al. 2021a, b). Therefore, some of the context related to the global feature, such as the curves used in the present study, may contribute to haptic roughness estimations.

There are still some aspects worthy of further research. In Experiment 5, we explored the effect of the global feature on the roughness of a fine surface. However, in the experiment, we only had two kinds of fine surfaces and found a significant interaction between surface features and roughness perception, which indicates that the degree of local features seems to determine the influence of global features on roughness perception. Further studies may require the inclusion of more types of fine surfaces and more levels of local features to address this problem. Moreover, in the present study, we only focused on the interaction between global and local information (different dimensions). The integration of information from different modalities (auditory or visual information), especially for indirect touch through a probe, is also worthy of further research (Jousmäki and Hari 1998). In addition, only the effects of local and global features on roughness were explored in this experiment. However, this investigation did not focus on the participants’ direct evaluation of physical factures, for instance, participants did not report feeling of spatial density in present study. This information would have given us a deeper understanding of the relationship between roughness perception and physical features. Subsequent neuroimaging studies may provide more evidence that could elucidate the underlying mechanism regarding haptic roughness perception.
