# Correction to: Global surface features contribute to human haptic roughness estimations

**DOI:** 10.1007/s00221-022-06317-7

**Published:** 2022-02-23

**Authors:** Huazhi Li, Jiajia Yang, Yinghua Yu, Wu Wang, Yulong Liu, Mengni Zhou, Qingqing Li, Jingjing Yang, Shiping Shao, Satoshi Takahashi, Yoshimichi Ejima, Jinglong Wu

**Affiliations:** 1 Graduate School of Interdisciplinary Science and Engineering in Health Systems, Okayama University, 3-1-1 Tsushima-Naka, Kita-ku, Okayama, 700-8530 Japan; 2 Section On Functional Imaging Methods, National Institute of Mental Health, Bethesda, MD USA; 3 School of Mechatronical Engineering, Beijing Institute of Technology, Beijing, China; 4 School of Psychological and Cognitive Sciences, Peking University, Beijing, China; 5 Department of Teacher Education, Wenzhou University, Wenzhou, China; 6 School of Computer Science and Technology, Changchun University of Science and Technology, Changchun, China; 7 School of Social Welfare, Yonsei University, Seoul, Korea

## Correction to: Experimental Brain Research 10.1007/s00221-021-06289-0

The article “Global surface features contribute to human haptic roughness estimations”, written by Huazhi Li, Jiajia Yang, Yinghua Yu, Wu Wang, Yulong Liu, Mengni Zhou, Qingqing Li, Jingjing Yang, Shiping Shao, Satoshi Takahashi, Yoshimichi Ejima and Jinglong Wu., was originally published electronically on the publisher’s internet portal on 16 January 2022 without open access. With the author(s)’ decision to opt for Open Choice the copyright of the article changed to © The Author(s) 2022 and the article is forthwith distributed under a Creative Commons Attribution 4.0 International License, which permits use, sharing, adaptation, distribution and reproduction in any medium or format, as long as you give appropriate credit to the original author(s) and the source, provide a link to the Creative Commons licence, and indicate if changes were made. The images or other third party material in this article are included in the article’s Creative Commons licence, unless indicated otherwise in a credit line to the material. If material is not included in the article’s Creative Commons licence and your intended use is not permitted by statutory regulation or exceeds the permitted use, you will need to obtain permission directly from the copyright holder. To view a copy of this licence, visit http://creativecommons.org/licenses/by/4.0.

Open access funding enabled and organized by Projekt DEAL.

Original article has been updated.

